# Frontliners on the Move: A Quantitative Analysis of the Prevalence of COVID-19 Reinfection Among Healthcare Workers

**DOI:** 10.7759/cureus.24652

**Published:** 2022-05-01

**Authors:** Nithin C Kurra, Krithika Sriram, Nikhila Gandrakota, Jai Sivanandan Nagarajan, Sujoy Khasnavis, Manju Ramakrishnan, Suhani Dalal, Shayan A Irfan, Sarah Khan, Hariniska JK, Dhruv Patel, Gayathri Samudrala

**Affiliations:** 1 Department of Neurology, University of Nebraska Medical Center, Omaha, USA; 2 Hospital-based Medicine, University of Pretoria, Pretoria, ZAF; 3 Family Medicine, Emory University School of Medicine, Atlanta, USA; 4 Internal Medicine, SRM Medical College Hospital And Research Centre, Chennai, IND; 5 Internal Medicine, St. George's University School of Medicine, True Blue, GRD; 6 Medicine, SRM Medical College Hospital and Research Centre, Chennai, IND; 7 Internal Medicine, University of Medicine and Health Sciences, Basseterre, KNA; 8 Medicine, Dow University of Health Sciences, Karachi, PAK; 9 Medicine, Fatima Jinnah Medical University, Lahore, PAK; 10 Medicine, KAP Viswanatham Government Medical College, Trichy, IND; 11 Internal Medicine, Medical University of Gdańsk, Gdańsk, POL; 12 Medicine and Surgery, Dr. NTR University of Health Sciences, Vijayawada, IND; 13 Obstetrics and Gynecology, National Board of Examinations, New Delhi, IND

**Keywords:** covid-19 infection, front line workers, covid-19 in physicians, sars-cov-2 (severe acute respiratory syndrome coronavirus -2), prevalence, reinfection, healthcare workers, covid-19

## Abstract

This study was conducted to review relevant articles and demonstrate the prevalence of coronavirus disease 2019 (COVID-19) reinfection among healthcare workers (HCWs). A systemic search was conducted on PubMed and Medline from their inception to July 17, 2021. All statistical analyses were conducted using ReviewManager 5.4.1. Studies meeting the following inclusion criteria were selected: (a) articles having HCWs with COVID-19; (b) studies describing reinfection of COVID-19; and (c) articles having a defined number of patients and controls. Three studies were selected for meta-analysis. The Newcastle-Ottawa Scale (NOS) was used to assess the quality of the cohort studies. NOS scores of 1-5 were considered high risk for bias, scores of 6-7 were deemed moderate, and scores >7 were considered low risk for bias. A random-effect model was used when heterogeneity was seen to pool the studies, and the results were reported in inverse variance (IV) and corresponding 95% confidence interval (CI). Pooled prevalence of reinfection of COVID-19 in HCWs was 3% (OR: 0.03 [-0.04, 0.01]; p=0.44;* I^2^*=4%). A non-significant prevalence was found among the healthcare professionals in terms of severe acute respiratory syndrome coronavirus 2 (SARS-CoV-2) reinfection in Europe. The preformed antibodies were protective against reinfection. However, the waning of antibodies with respect to time was evident, varying differently in different individuals, thereby resulting in reinfection.

## Introduction and background

Coronaviruses, a derivative of the RNA lineage, comprise a large family of viruses that cause respiratory infections. Coronavirus disease 2019 (COVID-19), caused by the novel severe acute respiratory syndrome coronavirus 2 (SARS-CoV-2), was declared a global pandemic in March 2020 by the World Health Organization (WHO), and it is the first time that the world has encountered a viral pandemic of such unprecedented magnitude, resulting in alarming outbreaks across 114 countries [1]. Over 500 million confirmed cases (512,282,384) of COVID-19 have been reported globally with over six million deaths (6,256,602) [2].

Depending on the type of strain and the patient's baseline immunity, clinical presentations of COVID-19 tend to vary from mild to severe. The most commonly reported symptoms include fever, cough, shortness of breath, fatigue, myalgia, sore throat, congestion, nausea, vomiting, diarrhea, and loss of taste or smell [3]. Biochemical profiles demonstrating lymphopenia, eosinopenia, thrombopenia, increased D-dimer levels, lactate dehydrogenase, C-reactive protein, troponins, and low zinc levels are associated with the severity of the disease [4]. It is postulated that SARS-CoV-2 binds to angiotensin-converting enzyme 2 (ACE2) receptors that are situated in both pulmonary and extrapulmonary sites, leading to endothelial cell damage after the overactivation of the inflammatory cascade. This occurs in parallel to varying degrees of dysregulation of the renin-angiotensin-aldosterone system and immune response [5-7].

To create robust diagnostic methods for the detection of COVID-19, several laboratories worldwide have worked on decoding the viral genome. Eventually, the first SARS-CoV-2 genomic sequence was published in January 2020 (GenBank accession number MN908947) [8]. Using this vital information, several testing modalities have now been adopted. For initial diagnostic testing, real-time polymerase chain reaction (RT-PCR) is the preferred first-line assay, whereas to detect levels of active virus, the nucleic acid amplification test (NAAT) is the technique of choice. In the former, PCR assay targets various components of the SARS-CoV-2 virus, which includes the envelope (E), nucleocapsid (N), and spike (S) genes, regions in the first open reading frame (orf1a and orf1b), and the RNA-dependent RNA polymerase (RdRp) gene [9,10]. Other techniques such as antigen detection, which include lateral flow sandwich immunoassays, chromatographic digital immunoassays, microfluidic immunofluorescence assays, and antibody detection, which target two SARS-CoV-2 antigens, the nucleocapsid (N), or spike (S) protein, and point-of-care tests are also currently being used for detection [11].

Several cases of COVID-19 reinfection have been reported [12-16]. Torres et al. presented a case of reinfection in a patient with more aggressive symptoms three months following the primary infection [12]. In this case, the IgA for COVID-19 was detected on ELISA. Loh et al. reported another case of a patient with X-linked agammaglobulinemia and bronchiectasis, three weeks after the primary infection [17]. Azam et al. conducted a meta-analysis on the prevalence of COVID-19 recurrence involving patients from China and Brunei only [18]. Arafkas et al. have analyzed data mostly related to case reports and cumulative cases, concluding that any observed COVID-19 relapse within a 90-day period might be a case of protracted primary infection rather than reinfection [19].

With the emergence of new variants of COVID-19 - Alpha, Beta, Gamma, Delta, and Omicron - the prevalence of reinfection might be affected as well [20]. In light of this, we conducted a meta-analysis highlighting the prevalence of COVID-19 reinfection among healthcare workers (HCWs).

## Review

Method

Data Sources and Search Strategy

This systematic review and meta-analysis was conducted according to the Preferred Reporting Items for Systematic Review and Meta analyses (PRISMA) guidelines [21]. An electronic search on PubMed and Medline was conducted from their inception to July 17, 2021 (detailed strategy provided in Table 1), using the search string: (("sars cov 2"[MeSH Terms] OR "sars cov 2"[All Fields] OR "covid"[All Fields] OR "covid 19"[MeSH Terms] OR "covid 19"[All Fields]) AND ("reinfect"[All Fields] OR "reinfecting"[All Fields] OR "reinfection"[MeSH Terms] OR "reinfection"[All Fields] OR "reinfected"[All Fields] OR "reinfections"[All Fields] OR "reinfects"[All Fields])) AND ((ffrft[Filter]) AND (clinicaltrial[Filter] OR journalarticle[Filter] OR meta-analysis[Filter] OR randomizedcontrolledtrial[Filter] OR review[Filter] OR systematicreview[Filter]) AND (fft[Filter]) AND (2019:3000/12/12[pdat]) AND (english[Filter])). In addition, we manually screened the cited articles for previous meta-analyses, randomized controlled trials, cohort studies, and review articles to identify any relevant studies.

**Table 1:** Search strategy

Search engine	Search strategy
Pubmed/Medline	(("sars cov 2"[MeSH Terms] OR "sars cov 2"[All Fields] OR "covid"[All Fields] OR "covid 19"[MeSH Terms] OR "covid 19"[All Fields]) AND ("reinfect"[All Fields] OR "reinfecting"[All Fields] OR "reinfection"[MeSH Terms] OR "reinfection"[All Fields] OR "reinfected"[All Fields] OR "reinfections"[All Fields] OR "reinfects"[All Fields])) AND ((ffrft[Filter]) AND (clinicaltrial[Filter] OR journalarticle[Filter] OR meta-analysis[Filter] OR randomizedcontrolledtrial[Filter] OR review[Filter] OR systematicreview[Filter]) AND (fft[Filter]) AND (2019:3000/12/12[pdat]) AND (english[Filter]))

Study Selection

Studies that met the following eligibility criteria were included: (a) articles having HCWs with COVID-19; (b) studies describing reinfection of COVID-19; and (c) articles having a defined number of patients and controls. Furthermore, the strategy adopted for the research can be defined as PECOS: 1) P (Population): COVID-19 patients; 2) E (Exposure): previous episode of COVID-19; 3) C (Control): no reinfection; 4) O (Outcome): the second episode of COVID-19; 5) S (Studies): human-based randomized controlled trials and cohort studies published in English only.

Statistical Analysis

ReviewManager (version 5.4.1; The Nordic Cochrane Centre, Copenhagen, Denmark, The Cochrane Collaboration, 2020) was used for all statistical analyses. The data from studies were pooled using a random-effects model when heterogeneity was seen. Analysis of results was done by calculating the inverse variance (IV) with respective 95% confidence intervals (CI). The chi-square test was performed to assess any differences between the subgroups. Sensitivity analysis was done to see if any individual study was driving the results and to ascertain reasons for high heterogeneity. As per the Cochrane handbook, the scale for heterogeneity was determined as follows: I^2^=25-60% - moderate; 50-90% - substantial; 75-100% - considerable heterogeneity, and p<0.1 indicated significant heterogeneity [22]. A p<0.05 was considered significant for all analyses.

Prevalence was calculated through raw data. This along with other extracted information was used to find standard errors using the following formula:

$SE=\sqrt{\frac{p\times \left ( 1-p \right )}{n}}$

Where “p” was the prevalence and “n” was the number of COVID-19 patients. The prevalence and standard error of each study were then input into the ReviewManager through the IV method in order to compute pooled prevalence along with a 95% CI.

Data Extraction and Quality Assessment of Studies

An independent search of electronic databases was done. Studies searched were exported to the EndNote Reference Library software version 20.0.1 (Clarivate, Philadelphia, PA), and duplicates were screened and removed.

Data extraction and quality assessment of included studies were done simultaneously. The Newcastle-Ottawa Scale (NOS) was used to assess the quality of the cohort studies. NOS scores of 1-5 were considered high risk for bias, scores of 6-7 were deemed moderate, and scores >7 were considered low risk for bias. Details of the scoring are provided in Table 2.

**Table 2:** Quality assessment of cohorts using Newcastle-Ottawa Scale (NOS)

Studies	Selection (maximum 4)				Comparability (maximum 2)	Outcome (maximum 3)			Total score
	Representativeness of the exposed cohort	Selection of the non-exposed cohort	Ascertainment of exposure	Demonstration that the outcome of interest was not present at the start of the study	Comparability of cohorts on the basis of the design or analysis	Assessment of outcome	Was follow-up long enough for outcomes to occur?	Adequacy of follow-up of cohorts	
Lumley et al. [23]	1	1	1	0	2	1	1	1	8
Sánchez-Montalvá et al. [24]	1	1	1	1	1	1	1	1	8
Hall et al. [25]	1	1	1	1	2	1	1	1	9

Results

Literature Search Results

**Figure 1:**
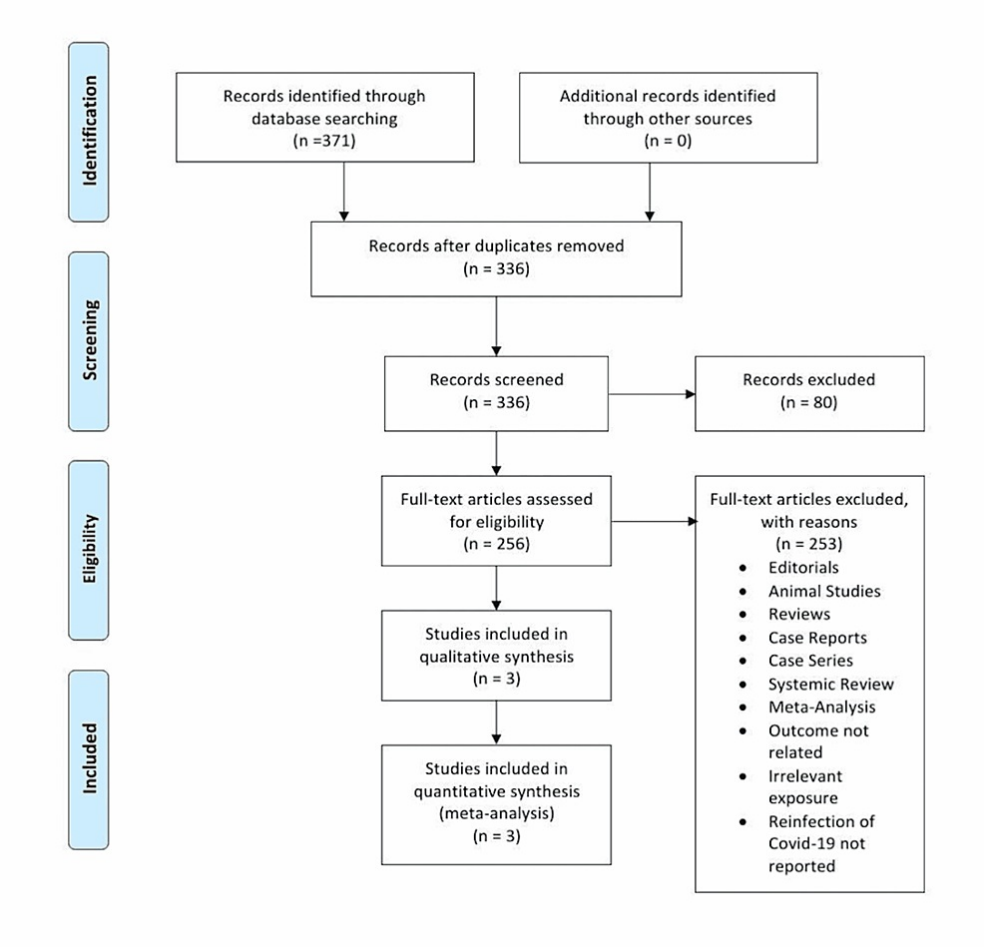
Flow chart of the selection of studies

The initial search of the electronic database yielded 371 potential studies. After exclusions based on titles and abstracts, the full texts of 256 studies were examined for possible inclusion. Current analyses of the available literature yielded 371 potential COVID-19 reinfection studies. After exclusions based on title and abstract screening, a full-text review of 256 studies was conducted for possible inclusion. Some of these studies focused on the presence of antibodies and their durability after COVID-19 infection, while some studies concentrated on the efficacy of serological testing in predicting reinfection chances. In addition, a couple of studies were ongoing longitudinal cohort studies with the goal of following seropositive and seronegative HCWs through 2022. However, many of these studies were only recently published. A total of three studies were ultimately selected for quantitative analysis [23-25] to assess the prevalence of COVID-19 recurrence after primary infection. Figure 1 summarizes the results of our literature search.

Study Characteristics

Table 3 provides the basic characteristics of the included studies. The three studies included a total of 26,153 participants. Table 4 summarizes the results of each study.

**Table 3:** Characteristics of the included studies HCWs: healthcare workers

Study name	Year	Study design	Duration	Country	Total HCWs (n)	Males (%)	Mean age (years)	Prevalence of reinfection (%)	Quality score
Lumley et al. [23]	2021	Cohort	April 23, 2020 - October 2020	United Kingdom	452	21	45.3	34.1	8
Sánchez-Montalvá et al. [24]	2021	Cohort	March 2020 - September 2020	Spain	40	45	30.5	2.86	8
Hall et al. [25]	2021	Cohort	June 18, 2020 - January 11, 2021	United Kingdom	25,661	15.6	44.9	1.9	9

**Table 4:** Analytical details of the selected studies CI: confidence interval

Study name and year		Reinfection of COVID-19		Odds ratio [95% CI]	P-value
		Prevalence (n)	Standard error (n)		
Lumley et al. [23]	2021	0.341	0.2229	0.34 [-0.10, 0.78]	0.1261
Sánchez-Montalvá et al. [24]	2021	0.0286	0.2635	0.03 [-0.49, 0.55]	0.9136
Hall et al. [25]	2021	0.019	0.0043	0.02 [0.01, 0.03]	0.0000

Publication Bias Assessment

Publication bias was not assessed since our review included only three studies. Studies used in this systematic review are not representative of the whole HCW population.

Results of Meta-Analysis

**Figure 2:**
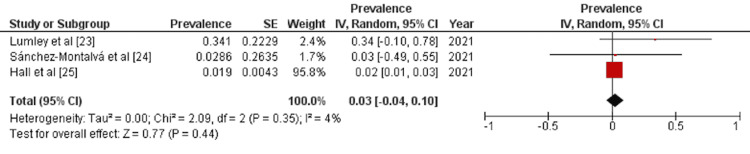
Forest plot showing effect size of the prevalence of reinfection of COVID-19 IV: inverse variance; CI: confidence interval

A detailed forest plot, outlining the effect size of the prevalence of reinfection of COVID-19, is illustrated in Figure 2. Pooled result showed statistically non-significant prevalence of reinfection of COVID-19 (OR: 0.03 [-0.04, 0.01]; p=0.44; I^2^=4%).

Discussion

In this meta-analysis, we evaluated the prevalence of COVID-19 recurrence after primary infection. Data were collected from HCWs in Spain and the United Kingdom. The analyzed data from three studies (n=26,153) showed that the pooled prevalence of COVID-19 reinfection in HCWs was 3% (OR: 0.03 [-0.04, 0.01]) and statistically non-significant (p=0.44).

Lumley et al. [23] presented the data of 452 HCWs with a mean age of 41 years, from April 2020 to October 2020. Their seroprevalence study measured SARS-CoV-2 anti-nucleocapsid and anti-spike IG, reporting a 34.1% prevalence of reinfection. Of note, 95 previously positive HCWs with positive SARS-CoV-2 anti-nucleocapsid IgG were symptomatic; 59 HCWs with positive antibodies were asymptomatic [23]. Sánchez-Montalvá et al. [24] presented the data from March 2020 to September 2020, of HCWs with a mean age of 29 years. The nasopharyngeal swab was used to detect the viral DNA, which utilizes transcription-mediated amplification (TMA) for nucleic acid amplification along with RT-PCR. They found that the rate of incidence for reinfection was 28.6 cases per 1,000 person-week [24]. Hall et al. [25] included HCWs with a median age of 45.6 years; for detecting reinfection, NAAT with RT-PCR was used at regular intervals of two weeks for seven months, along with a SARS-CoV-2 antibody test. Among the 155 HCWs from a positive cohort population of 8,278, 50 HCWs were symptomatic of COVID-19 (six per 1,000 HCWs), 28 HCWs showed other symptoms (3.4 per 1,000 HCWS), and 76 were asymptomatic (9.2 per 1,000 HCWS), and there were two probable cases. Of note, 81.9% of the HCWs were antibody-positive; 71.8% had symptoms, and 100% were probable cases, whereas 1,704 new PCR-positive infections were reported from a negative cohort population of 17,383. The incidence in the positive cohort compared to the negative cohort was 7.6 reinfections to 57.3 primary infections per 100,000 person-days [25].

A previously published meta-analysis by Azam et al. showed significant results for reinfection, with a prevalence of 14.81%, primarily detected through PCR, predominantly involving the Chinese population [18]. According to Dao et al., several factors such as false RT-PCR results, viral reactivation or reinfection with another SAR-CoV-2 strain, or intermittent viral shedding can contribute to false-positive RT-PCR results [26]. False-negative RT-PCR results leading to patient discharge during primary infection are also a possibility [26]. Several reasons for false RT-PCR have been postulated, mainly poor-quality sampling and insufficient quantity of cellular material on the swab [27]. Thermal inactivation was also found to decrease the sensitivity of RT-PCR [28]. The rate of false-negative RT-PCR varies from 3 to 41%, varying according to the type of clinical specimen used [29]. Feng et al. presented a case of a false-negative found on four sequential RT-PCR tests for COVID-19, with multifocal ground-glass opacities on the left upper lobe of the patient’s lung. The infection was ultimately detected using the fifth RT-PCR test [30]. Arafkas et al., in their meta-analysis, found no case report with clinical reinfection after a 70-day period following primary infection [19].

Bao et al., in their experiment on Rhesus macaques, found protective effects of primary infection from subsequent exposures in monkeys [31]. The immune response of the COVID-19 patients is variable and patient-specific with respect to antibody development, with variations in the persistence of antibodies in serum [18]. The anti-SARS-CoV-2 IgG was found positive in more than 95% of patients following primary infection [32,33]. After a month from initial infection, IgG and IgM antibodies are highest in concentration; however, levels do not increase after post-negative positive RT-PCR results, suggesting positive RT-PCR results via the detection of RNA particles rather than reinfection [34,35]. Krutikov et al. conducted a study on staff and residents from 100 long-term facilities, with age <65 years among staff and age >65 years among residents; they reported that although staff and residents with antibodies against the SARS-CoV-2 nucleocapsid protein at baseline remain susceptible to symptomatic infection, their risk of reinfection is low (<1% risk per month) for up to 10 months after primary reinfection [36]. Post et al. showed evidence of waning of antibody titers over time [32]. The waning of antibodies results in compromised immunity against COVID-19 [37]. Based on the evidence in the literature, reinfection is found relatively more prevalent in immunocompromised patients [17,38,39].

Based on these findings, the reinfection of SARS-CoV-2 is not prevalent in HCWs; however, evidence in the literature suggests a significant chance of reinfection in immunocompromised patients or patients with a decrease in antibodies with respect to time. The variation in viral stain might be a contributing factor in reinfection; however, no prominent evidence has been reported. Reduced sensitivity of delta SARS-CoV-2 variant against antibody neutralization was reported by Planas et al. [40]. To determine COVID-19 reinfection among HCWs, new studies should be conducted as the presented data is not sufficient to predict any significant results.

Limitations

Our study is limited by certain factors, such as (a) all studies were cohort in nature, (b) only a few studies were available, (c) the total population was not sufficient considering the enormity of the pandemic, (d) studies with different settings were pooled, (e) imputation method was used to put standard error, which is provided in methods, (f) these studies were pivotal in informing our analysis, but more studies that are community-based and with random controls should be conducted, and (g) studies used in this systematic review are not representative of the whole HCW population.

## Conclusions

A non-significant prevalence was found among the healthcare professionals in terms of SAR-CoV-2 reinfection in Europe. It is evident that the preformed antibodies are protective against reinfection. However, over time, the apparent waning of antibodies might result in recurrence, depending on the varying neutralizing antibody levels in different individuals. Evidence in the literature also suggests that immunocompromised patients are at a higher risk of reinfection. The presented data is not sufficient to predict any significant results; there is a need for further research with the emergence of new COVID-19 variants. At this stage, the natural immunity from COVID-19 infection or the vaccine and public health measures are the primary approaches to prevent this course. Therefore, due to the observed low prevalence of reinfection, the focus should be placed on addressing vaccine hesitancy and establishing wide coverage of COVID-19 vaccination to limit the spread of new infections among HCWs.
